# Applying a Women’s Health Lens to the Study of the Aging Brain

**DOI:** 10.3389/fnhum.2019.00224

**Published:** 2019-07-05

**Authors:** Caitlin M. Taylor, Laura Pritschet, Shuying Yu, Emily G. Jacobs

**Affiliations:** ^1^Department of Psychological and Brain Sciences, University of California, Santa Barbara, Santa Barbara, CA, United States; ^2^The Sage Center for the Study of the Mind, University of California, Santa Barbara, Santa Barbara, CA, United States; ^3^Neuroscience Research Institute, University of California, Santa Barbara, Santa Barbara, CA, United States

**Keywords:** cognitive aging, neuroimaging, women’s health, sex steroid hormones, estradiol, menopause, reproductive aging, memory

## Abstract

A major challenge in neuroscience is to understand what happens to a brain as it ages. Such insights could make it possible to distinguish between individuals who will undergo typical aging and those at risk for neurodegenerative disease. Over the last quarter century, thousands of human brain imaging studies have probed the neural basis of age-related cognitive decline. “Aging” studies generally enroll adults over the age of 65, a historical precedent rooted in the average age of retirement. A consequence of this research tradition is that it overlooks one of the most significant neuroendocrine changes in a woman’s life: the transition to menopause. The menopausal transition is marked by an overall decline in ovarian sex steroid production—up to 90% in the case of estradiol—a dramatic endocrine change that impacts multiple biological systems, including the brain. Despite sex differences in the risk for dementia, the influence that biological sex and sex hormones have on the aging brain is historically understudied, leaving a critical gap in our understanding of the aging process. In this *Perspective* article, we highlight the influence that endocrine factors have on the aging brain. We devote particular attention to the neural and cognitive changes that unfold in the middle decade of life, as a function of reproductive aging. We then consider emerging evidence from animal and human studies that other endocrine factors occurring earlier in life (e.g., pregnancy, hormonal birth control use) also shape the aging process. Applying a women’s health lens to the study of the aging brain will advance knowledge of the neuroendocrine basis of cognitive aging and ensure that men and women get the full benefit of our research efforts.

## Introduction

An overarching goal of cognitive neuroscience is to understand the complexities of human brain function across the lifespan. To make sense of cognition and behavior, scientists test hypotheses on a “representative” sample of individuals that are assumed to generalize to a larger population. Here, we argue that it is imperative for scientists to reconsider what constitutes a representative sample.

A pressing problem in the biomedical sciences is the under-representation of females in experimental designs. For the past half-century, the convention in preclinical research has been to study male animals, at the near-exclusion of females. Females were considered “too variable” (Beery and Zucker, 2011) despite empirical evidence that variability within each sex is the same across a broad range of phenotypes (Prendergast et al., 2014). In 2016 in the US, this sex bias in biomedical research was addressed at the national level when Janine Clayton (Director, Office of Women’s Health Research) pioneered the National Institute of Health’s mandate requiring the inclusion of female and male animals in preclinical science (Clayton and Collins, 2014). The goal of the mandate is to ensure that future studies are balanced by sex, a key step to bolstering our understanding of sex similarities and sex differences across the biomedical sciences.

In human neuroscience, the problem is more subtle. While the majority of studies enroll both men and women, women do not benefit equally from our research efforts. Scientists often overlook sex-specific variables, a bias that seeps into our study design and analyses and impedes our basic understanding of the brain. In this *Perspective* article, we address one domain that is often unaccounted for in human neuroscience: the influence of sex steroid hormones on the brain. This is surprising, given that the brain is an endocrine organ and in animal studies, the effects of sex hormones on the central nervous system are extensive, ranging from changes in gene expression to alterations in behavior (McEwen, 2001). Across a typical menstrual cycle (occurring every 25–30 days), naturally cycling women experience a ~12-fold increase in estrogen and an ~800-fold increase in progesterone. Later in life, women experience a more abrupt change in sex steroid hormone production as they transition through menopause. Further, sex hormone production is chronically suppressed in the 100 million women worldwide using oral hormonal contraceptives (OCs). For men, testosterone production shows a gradual, protracted decline beginning in the mid-30s and continuing throughout life. How do these shifts in hormone production shape the brain? Do endocrine factors influence how the brain ages? The field of human neuroscience has not adequately addressed these questions and women may be disproportionately disadvantaged by this oversight.

Below, we describe animal and human evidence that sex hormones regulate the structure and function of brain regions critical to learning and memory. We focus on the implications of this work for understanding the neurobiological mechanisms of cognitive aging. Moving forward, the field of human cognitive neuroscience must consider features (e.g., the menstrual cycle, menopause, pregnancy, and OC use) that are relevant to half of our study population. If not, we will be left with an inadequate understanding of the aging brain and will risk the health of half of the world’s population.

## The Neuroendocrine Basis of Cognitive Aging

A major challenge in neuroscience is to understand what happens to a brain as it ages. Distinguishing between individuals who undergo typical aging from those at risk for neurodegenerative disease is critical for targeting early interventions to high-risk individuals. Over the last quarter century, thousands of human brain imaging studies have probed the neural basis of age-related cognitive decline. These studies generally enroll adults over the age of 65, a historical precedent rooted in the average age of retirement. A consequence of this research tradition is that it overlooks one of the most significant neuroendocrine changes in a woman’s life: the transition to menopause. The menopausal transition is marked by an overall decline in ovarian sex steroid production—up to 90% in the case of estradiol—a dramatic endocrine change that impacts multiple biological systems, including the brain (Morrison et al., 2006).

**Figure 1:**
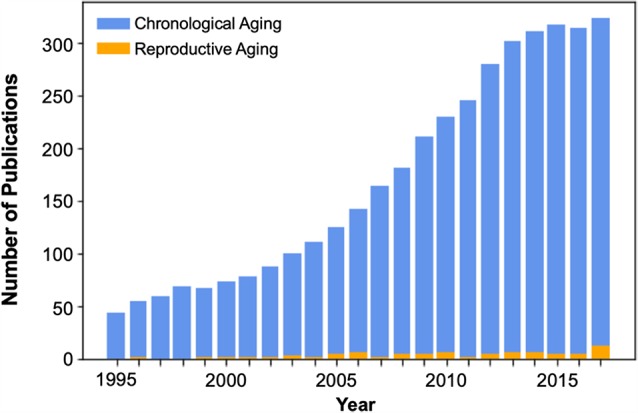
Publication count of cognitive neuroscience studies of aging, beginning in the mid-1990s with the widespread adoption of functional brain imaging techniques. The number of brain imaging publications that consider the effects of reproductive or “neuroendocrine” aging during the midlife transition to menopause is dwarfed by the number of chronological aging studies, which compare men and women >65 to young adults. Over the past 23 years there have been only 82 brain imaging publications on reproductive aging. Of those, only 49% used endocrine assessments to verify menopausal stage (see Supplementary Material).

In the context of cognitive aging, female reproductive aging presents a critical yet understudied factor (Figure 1) that is likely essential for understanding the early processes that contribute to age-related cognitive decline and ultimately dementia risk. Indeed, growing evidence from animal studies indicates that sex steroids including estradiol, progesterone, and testosterone, play a substantial role in supporting the structure and function of brain regions relevant to cognitive aging (Jacobs and Goldstein, 2018).

### Sex Hormone Action in Memory Circuitry

The actions of estrogen in the brain are in large part dependent on the location of estrogen receptors (ERs; McEwen and Alves, 1999). At the cellular level, estrogen, primarily in the form of 17β-estradiol, facilitates synaptogenesis, protects against oxidative stress, and regulates neuromodulators including serotonin, norepinephrine, dopamine, and acetylcholine (Becker, 1990; Thompson and Moss, 1994; McEwen and Alves, 1999; McEwen et al., 1997; McEwen and Alves, 1999; Walf and Frye, 2006; Wang et al., 2010; Chisholm and Juraska, 2012; Bean et al., 2014; Galvin and Ninan, 2014; Almey et al., 2015; Hara et al., 2015, 2016; Rossetti et al., 2016; Frick et al., 2018).

Estradiol signaling is a critical component of cell survival and plasticity, and its effects can be measured across multiple spatial and temporal scales (Frick et al., 2018). Many of these effects occur in brain regions that are critical to higher level cognitive function and cognitive aging. In non-human primates, at the cellular level, nearly 50% of prefrontal cortex (PFC) pyramidal neurons express the ERα subtype (Wang et al., 2010), and greater ERα expression is associated with better short-term memory performance (Wang et al., 2010). Further, the suppression of ovarian hormones decreases spine density in PFC neurons (Hao et al., 2006), and impairs working memory performance (Rapp et al., 2003). In rodents, in the hippocampus, dendritic spine density in CA1 neurons varies over the course of the estrous cycle (Woolley et al., 1990; Woolley and McEwen, 1993). At the macroscopic level, hippocampal volume is regulated by sex hormones (Galea et al., 1999) and fluctuates across the estrous cycle (Qiu et al., 2013). These basic science findings provide converging evidence that the manipulation of estrogen levels leads to structural and functional changes in the ER-enriched regions that comprise memory circuitry.

Human studies further implicate sex steroids in the regulation of memory circuitry (Berman et al., 1997; Shaywitz et al., 1999; Jacobs and D’Esposito, 2011; Epperson et al., 2012; Hampson and Morley, 2013; Shanmugan and Epperson, 2014; Jacobs et al., 2015, 2016, 2017; Albert et al., 2017; Girard et al., 2017; Zeydan et al., 2019), yet despite this evidence the neuroendocrine basis of cognitive aging remains understudied in human neuroscience.

## Menopause and Hormone Therapy

One of the most significant neuroendocrine changes in a woman’s life is the transition to menopause, during which circulating ovarian hormone concentrations decline up to 90%. Many women report changes in memory and attention (e.g., “menopause fog”) during this transitional period (Greendale et al., 2011). The median age of menopause is 52.4 years (Gold et al., 2001), yet the vast majority of cognitive aging studies target adults age 65 and older, missing this critical midlife window (Figure 1). The field has focused almost exclusively on the neural and cognitive effects of chronological aging, overlooking the impact of reproductive aging. This is striking, given that most women will spend one-third of their lives in the post-reproductive years, and mounting evidence suggests that reproductive aging influences brain structure, function, and cognition.

A significant methodological and ethical challenge of studying menopause is that to fully understand the impacts of this major hormonal shift on the brain, our designs must parse the parallel and interactional effects of chronological and reproductive aging in women. In humans, the relationship between gonadal aging and the brain has typically been studied in two contexts: studying the effects of spontaneous menopause and surgical menopause (e.g., bilateral salpingo-oophorectomy prior to natural menopause). For longitudinal studies of women experiencing spontaneous menopause, the effects of chronological and reproductive aging cannot be separated. However, in studying women who have undergone surgical menopause or cross-sectional studies that pair age-matched women who fall within different stages of the menopausal transition, the effects of reproductive aging alone can be more effectively characterized.

### Impact of Gonadectomy and Hormone Supplementation in Animals

While challenging in humans, animal studies more easily decouple the effects of reproductive aging from chronological aging *via* surgical menopause (gonadectomy) paradigms. These studies demonstrate that ovarian hormone depletion impacts hippocampal and PFC morphology and function, independent of the established influence of chronological aging. This body of work has made significant progress toward characterizing the synaptic basis of menopause-related memory decline (Morrison and Baxter, 2012; Hara et al., 2016). For example, rodent and nonhuman primate studies first identified estradiol’s role in modulating structural plasticity in the hippocampus and PFC as well as estradiol’s protective effects against cognitive decline (Morrison and Baxter, 2012; Hara et al., 2015, 2016). In female macaques, surgical menopause leads to a 30% loss in spine density in hippocampal CA1 neurons, which is reversed by estradiol replacement (Dumitriu et al., 2010). Natural menopause in rhesus monkeys reduces the density of perforated synapse spines in CA1 neurons, which is correlated with lower recognition memory (Hara et al., 2012). Cyclic estradiol administration in postmenopausal female monkeys restores dorsolateral PFC spine density and the frequency of multisynaptic boutons to levels comparable to premenopausal females, and these synaptic-level changes are accompanied by enhanced performance on PFC-dependent memory tasks in estradiol-treated animals (Hara et al., 2016; Kohama et al., 2016).

### Impact of Menopause and Hormone Supplementation in Humans

Epidemiological surveys indicate that many women report increased forgetfulness and “brain fog” during the menopausal transition (Greendale et al., 2011). Neuropsychological studies have identified decrements in verbal fluency and associative memory tied to reproductive stage (Epperson et al., 2013; Weber et al., 2014; Rentz et al., 2017), and across women higher estradiol levels are associated with better memory performance (Rentz et al., 2017).

At the level of functional brain networks, our group showed that PFC activity and working memory performance are modulated by endogenous estradiol concentrations (Jacobs and D’Esposito, 2011; Jacobs et al., 2017). Using a within-woman, repeated-measures approach that capitalizes on the natural fluctuations in estradiol over the menstrual cycle in premenopausal women, we found that PFC activity is exaggerated when estradiol concentrations are low, a putative marker of neural inefficiency (Jacobs and D’Esposito, 2011). This “inefficient” PFC response is also evident in midlife women as ovarian estradiol production declines during the menopausal transition (Jacobs et al., 2016). In another population-based functional magnetic resonance imaging (fMRI) study, midlife men and women (*N* = 200; age range: 45–55) performed a verbal memory encoding task. Task-evoked hippocampal responses differed by women’s reproductive stage, despite minimal difference in chronological age. Across women, lower estradiol concentrations were related to greater alterations of hippocampal connectivity and poorer performance on a subsequent memory retrieval task, implicating sex steroids in the regulation of memory circuitry (Jacobs et al., 2016, 2017). Thus, early functional changes in memory circuitry are evident decades before the age-range typically targeted by cognitive neuroscience studies of the aging brain.

At the level of brain morphology, Zeydan et al. (2019) found that abrupt hormonal changes associated with early surgical menopause lead to structural abnormalities in the medial temporal lobe. The parahippocampus-entorhinal cortex was thinner in women who underwent bilateral ovariectomization compared to an age-matched premenopausal control group, despite the use of estrogen replacement in the surgical menopause group. Future studies should employ high resolution hippocampal subfield imaging to identify the impact of hormone suppression within specific medial temporal lobe structures, particularly subfields that may differ by cytoarchitecture and magnitude of ERα- and ERβ-expression.

A handful of human studies have directly examined the effect of hormone therapy (HT) on brain morphology in peri/postmenopausal women, revealing that hippocampal volume increases in response to certain hormone replacement regimens (Albert et al., 2017). The macrostructural changes evident in the hippocampus in response to estradiol supplementation may produce cognitive benefits (for a review, see Daniel et al., 2015). For example, Maki et al. (2011) found that women who began HT in perimenopause had enhanced hippocampal activity during a verbal recognition task and better verbal memory performance relative to nonusers. When initiated early in the menopausal transition, HT also appears to enhance cognitive control-related dorsolateral PFC activity and improve task-switching performance in women (Girard et al., 2017).

Together, these findings underscore the importance of considering reproductive stage, not simply chronological age, to identify neural and cognitive changes that unfold in the middle decade of life. In keeping with animal evidence, human studies demonstrate that the decline in ovarian estradiol production during menopause plays a role in shaping the structure and function of brain networks that support higher-order cognitive functions.

## Pregnancy

With a global fertility rate of 2.5 births per woman (World Bank, 2017), the majority of women will experience pregnancy at least once in their lifetime. Pregnancy is another prolonged period of major hormonal change, during which women experience a dramatic rise in sex steroid hormone concentrations. For instance, in humans, estradiol and progesterone levels increase up to 300-fold across the 40-week gestational period (Berg and Kuss, 1992; Tal et al., 2000; Schock et al., 2016), with progesterone levels rising from a mean of 1 ng/mL during an average menstrual cycle to 100–300 ng/mL during the last trimester of pregnancy (Tal et al., 2000; Schock et al., 2016). In rodents, this sustained increase in hormone levels during gestation has a lasting impact on the brain, particularly regarding hippocampal plasticity (reviewed in Kinsley and Lambert, 2008; Workman et al., 2012; Galea et al., 2014).

### Impact of Pregnancy on Brain Structure/Function in Animals

In rodents, the reproductive experience (i.e., pregnancy, lactation, and parenting) affects hippocampal morphology (Kinsley et al., 2006; Pawluski and Galea, 2006, 2007; Barha et al., 2015). Hippocampal CA1 spine density is significantly higher in late pregnancy and lactating females compared to nulliparous female rats at any phase of the estrous cycle (Kinsley et al., 2006). Pregnancy also affects long-term hippocampal sensitivity to estrogen (Roes and Galea, 2016). Barha and Galea (2011) studied hippocampal sensitivity to estrogens (17β, 17α, and estrone) in middle-aged rats as a function of parity (multiparous vs. nulliparous). All estrogens induced upregulation of cell proliferation in the hippocampus in multiparous females, however, none of the estrogens induced proliferation in nulliparous females. Further, pregnancy’s effects appear to be cumulative, such that the effects of pregnancy compound with subsequent parity. In a study of spatial learning and memory, Gatewood et al. (2005) found that multiparous rats exhibited better spatial learning and memory retention compared to age-matched primi- and nulliparous females when tested at 6, 12, 18, and 24 months of age. Additionally, immunohistochemistry within the CA1 region and dentate gyrus of the hippocampus of these rats revealed an effect of reproductive experience on amyloid precursor protein (APP) immunoreactive neurons. Multiparous females had fewer APP stained cells than primi- and nulliparous groups, and less APP staining corresponded with better behavioral performance at 24 months.

### Impact of Pregnancy on Brain Structure/Function in Humans

Pregnancy typically confers an enhancement of hippocampal-dependent memory in rodents, yet human studies report memory impairments during pregnancy. Similar to the self-reported cognitive changes experienced by menopausal women, pregnant women describe cognitive changes during pregnancy that include increased forgetfulness, greater distractibility, and word finding difficulties (reviewed in Brett and Baxendale, 2001). In a meta-analysis, Henry and Rendell (2007) observed that pregnant women exhibit impairments in free and delayed recall, subjective memory (persisting 3 months post-partum), and working memory relative to non-pregnant controls. Pregnant women did not outperform non-pregnant women in any domain. Glynn (2012) found that the effects on memory are cumulative with increasing parity. In the study, 254 women were evaluated on measures of verbal recall memory at four points during pregnancy and at 3 months post-partum. Beginning at 16 weeks’ gestation, the performance of women who had given birth more than twice was worse than the performance of women who had given birth once, which was worse than women who had not yet given birth (primigravid), with impairments persisting to 3 months post-partum.

Few neuroimaging studies have been conducted during pregnancy in humans. Limited findings suggest that global brain volume decreases during pregnancy (Oatridge et al., 2002) with an increase in brain volume after delivery (Kim et al., 2010), returning to pre-pregnancy levels by 24 weeks post-partum (Oatridge et al., 2002). Hoekzema et al. (2017) observed gray matter volume (GMV) reductions in primiparous women scanned before and after pregnancy. Reductions were observed across a network of regions that support social cognition and theory of mind, including the hippocampus, precuneus, and medial/inferior frontal gyrus. Gray matter volume reductions persisted up to 2 years post-partum (with some rebound in the hippocampus). The authors propose that these gray matter changes may facilitate the transition to motherhood, as the areas exhibiting volumetric reductions also exhibited the strongest fMRI BOLD response to pictures of the mothers’ infants compared to unrelated children. The magnitude of the morphological change (e.g., in cortical thickness, surface area, sulcal depth, etc.) in these mothers as a result of pregnancy were on par with the changes observed in adolescent males and females during the pubertal transition (Carmona et al., 2019).

### Association Between Pregnancy and Cognitive Aging

Greater lifetime exposure to estrogen is considered to be neuroprotective (Smith et al., 1999; Rasgon et al., 2005; Ryan et al., 2009; Heys et al., 2011; Tierney et al., 2013) and pregnancy has a lasting impact on circulating sex steroid hormones. Pregnancy appears to reduce lifetime estrogen exposure relative to nulliparity (summarized in Smith et al., 1999). Circulating estrogen levels are ~22% lower in parous women compared to nulliparous women (Bernstein et al., 1985). This difference persists through menopause, with 20% lower free estradiol levels in multiparous (≥4 children) compared to primiparous menopausal women (Chubak et al., 2004). This parity-related difference in hormone levels may contribute to findings that lower parity is associated with better postmenopausal cognitive function (McLay et al., 2003; Heys et al., 2011; but see Ryan et al., 2009; Tierney et al., 2013). Similarly, parity may have an effect on cognitive aging and dementia risk (reviewed in Roes and Galea, 2016), with reports that having children correlates with earlier onset of AD (Ptok et al., 2002) and a greater extent of AD pathology post-mortem (Beeri et al., 2009). Some of these effects are compounded by successive pregnancies (Sobow and Kloszewska, 2004; Colucci et al., 2006).

## Oral Hormonal Contraceptive Use

Ten million women in the US and 100 million women worldwide use oral hormonal contraception (OC; Petitti, 2003; Christin-Maitre, 2013; Jones et al., 2013; Daniels and Mosher, 2013; Daniels et al., 2015). First introduced in the US in 1960, “the pill” revolutionized women’s reproductive health. However, emerging evidence suggests that OCs influence aspects of brain structure and function in young adults (for review, see Pletzer and Kerschbaum, 2014). In two MRI studies, OC use in women was associated with increased GMV in the amygdala, parahippocampal gyrus (Pletzer et al., 2010; Lisofsky et al., 2016) and ventral temporal cortex (Pletzer et al., 2010, 2015) relative to non-users. Less robust effects have been observed in the PFC, although this finding is inconsistent across studies (Pletzer et al., 2010; De Bondt et al., 2013b; Petersen et al., 2015). Moving forward, the field would benefit from a well-powered study that can determine the influence of OC formulation, age of initiation, and duration of use on global and regional brain morphology.

No systematic study has been conducted to investigate the effects of chronic ovarian hormone suppression on brain regions that are densely populated with sex steroid receptors and are modulated by sex steroid hormones. Does long-term ovarian hormone suppression have consequences at the macroscopic level of regional brain morphology in humans? Are there enduring effects even after cessation of use? Though this area of research is understudied, retrospective studies suggest that OC use confers a positive effect on cognitive aging (Egan and Gleason, 2012; Karim et al., 2016). For instance, in an epidemiological study of postmenopausal women, Karim et al. (2016) found that hormonal contraceptive use was positively associated with global cognition and verbal memory. However, other studies report no relationship between OC use and cognitive outcomes (McLay et al., 2003; Tierney et al., 2013). The dearth of research on this topic is especially apparent when attempts are made to explain the endocrine basis of OC’s cognitive effects, with some studies attributing positive effects to the supraphysiological levels of synthetic sex hormones in OC users (Egan and Gleason, 2012; Karim et al., 2016), while other studies refer to suppressed levels of endogenous estrogen in OC users (Griksiene and Ruksenas, 2011; De Bondt et al., 2013a). Careful endocrine evaluations paired with studies that control for OC formulation are necessary to resolve these discrepancies.

In addition to OC formulation, the age of initiation of OC use must be considered. Up to one-third of OC users begin to use in early adolescence, yet we know relatively little about how hormone suppression impacts the developing brain, and this may be critical for understanding OC’s effects throughout the lifespan. While the hippocampus and basal ganglia typically reach adult levels in late childhood or early adolescence (Segawa, 2000; Gogtay et al., 2006), the development of the PFC is protracted, with cortical volumes stabilizing in the mid-20s (Lenroot and Giedd, 2006). The neuroendocrine changes that accompany puberty produce what has been referred to as a second “window of opportunity” or sensitive period in brain development (for review, see Fuhrmann et al., 2015). In females, the pubertal transition typically begins at 10–11 years of age and ends between the ages of 15–17. It is during this pubertal period that many women begin OC use. Among insured teenagers in the United States, 6% of 13-year-olds and 36% of 13–18-year-olds filled a prescription for OC in 2009 (Ehrlich et al., 2011). Given the early age of first exposure, OC use has the potential to alter the organizational effects of endogenous estradiol in adolescents through chronic suppression of sex steroid hormone levels. To our knowledge, no large-scale longitudinal study has examined the impact that age of initiation and duration of OC use have on neuronal development. Additionally, the short- and long-term effects of OC likely differ. In adults, short-term OC use is associated with GMV changes (Pletzer et al., 2015; Lisofsky et al., 2016), yet few studies have examined whether these changes persist over time, or whether the magnitude of GMV change correlates with total duration of use (Pletzer et al., 2010; De Bondt et al., 2013b; Petersen et al., 2015).

## Addressing the Under-Representation of Women’S Health Factors in Future Aging Studies

The biomedical sciences are witnessing a remarkable change, whereby researchers are recognizing the importance that sex plays in virtually all aspects of health and disease (Cahill, 2006; McCarthy, 2008). However, in neuroscience, the influence of biological sex and sex hormones on the aging brain remains understudied, leaving a critical gap in our understanding of the aging process. Researchers in the cognitive aging field often account for a variety of “lifespan” factors when characterizing their sample population (e.g., years of education, lifetime physical activity, history of smoking, or substance abuse), yet the endocrine lifespan is usually overlooked.

Recently there has been an appeal for earlier identification of individuals at risk for cognitive decline and dementia, with increasing focus on middle age. Yet few human studies have investigated the neurobiological and neuropsychological impact of reproductive aging, the onset of which coincides with this critical midlife window. Moving forward, the field should pay greater attention to the endocrine basis of brain aging by targeting under-represented samples of women, such as midlife women transitioning through menopause, women undergoing chronic hormone suppression for endocrine-related disorders like endometriosis, and women who undergo early surgical menopause. Enriching this area of research is sorely needed.

In addition to designing studies that address the needs of under-served populations, researchers can take a simpler step forward by adding a standardized reproductive health history questionnaire to their demographic batteries. This is particularly important for large-scale, publicly available data repositories that collect brain imaging and cognitive data on community-based cohorts (e.g., WU-Minn Human Connectome Project, Harvard Brain Genomics Superstruct Project, Philadelphia Neurodevelopmental Cohort). Few of these databases include standardized data on parity, use of hormone-based medications, menstrual cycle histories and/or incidence of common endocrine disorders. The Human Connectome Project-Aging makes progress on this front by collecting serum and saliva samples for hormone characterization and by using an enriched medical history questionnaire that includes some assessments of reproductive health (Bookheimer et al., 2019).

If the practice of collecting a standardized reproductive health history becomes routine, the field will be better able to incorporate hormone factors into models of the aging brain and can then use these findings to guide tightly controlled follow-up studies. Adopting this standard would provide a richer characterization of the sample population being studied and could enable meta-analyses that model the impact of endocrine variables on brain and cognitive outcomes. For example, do women who undergo early vs. late menopause show worse cognitive performance and greater neuropathology later in life? Does age of initiation or duration of OC use alter age-related changes in brain morphology? Do common medications that suppress sex hormone levels (e.g., Lupron for endometriosis) have enduring effects on brain structure, function, and cognition? Answers to these questions are long overdue.

## Conclusion

The biomedical sciences have treated the male as the representative sex for half a century. As Kathleen Okruhlik wrote in Okruhlik (1994):

“…the treatment of menstruation, pregnancy, and childbirth as diseases or medical emergencies may be traced to the fact that these are not things that happen to the ideal healthy human being who is, of course, male. The ideal healthy lab rat is also male. His body, his hormones, and his behaviors define the norm; so he is used in experiments. Female hormones and their effects are just nuisance variables that muck up the works, preventing experimenters from getting at the pure, clean, stripped-down essence of rat-hood as instantiated by the male model. Insofar as the female of the species is truly a rat (or truly a human being), she is covered by the research on males. Insofar as she is not included in that research, it is because she is not an archetypal member of her own species. The dangerous effects of such research procedures, especially in the biomedical sciences, are just now being documented. For far too long, the assumption underlying these experimental designs (that males are the norm) simply went unchallenged.”

Science has to represent society, especially since the bulk of academic research is publicly funded by tax-payer dollars. Moving forward, scientists must ensure that our research program serves men and women alike. Historically, cognitive neuroscience has largely overlooked aspects of the human condition (the menstrual cycle, OCs, pregnancy, menopause) that are relevant to half of the world’s population (and half of the US tax-base), and should correct course in order for the field to advance.

## Data Availability

No datasets were generated or analyzed for this study.

## Author Contributions

CT, LP and EJ wrote the manuscript. LP and SY edited the manuscript. SY conducted the literature survey provided in the supplemental figures.

## Conflict of Interest Statement

The authors declare that the research was conducted in the absence of any commercial or financial relationships that could be construed as a potential conflict of interest.
