# Supplementary material for: Applying a Women’s Health Lens to the Study of the Aging Brain
Source: Front Hum Neurosci. 2019 Jul 5;13:224. doi: 10.3389/fnhum.2019.00224 (PMC6625223; doi:10.3389/fnhum.2019.00224)
Supplement: Supplementary file 1 [file Data_Sheet_1.pdf]

Supplementary Material

Summary

Since the advent of neuroimaging, thousands of human brain imaging studies have sought to understand the neural basis of age-related cognitive decline. These studies have generally enrolled adults over the age of 65, which is a historical precedent rooted in the average age of retirement. A consequence of this practice is that studies of cognitive aging have largely overlooked one of the most significant neuroendocrine changes in a woman's life: the transition to menopause. The menopausal transition, typically occurring between ages 45-55, is marked by an almost total cessation of ovarian sex steroid production—up to 90% in the case of estradiol—a dramatic endocrine change that impacts multiple biological systems including the brain. In the context of cognitive aging, female reproductive aging represents a critical yet understudied factor that is likely essential for understanding the early processes that contribute to age-related cognitive decline and, ultimately, risk for dementia.

To understand the extent of this oversight, we sought to quantify the number of neuroimaging publications that examine the effects of reproductive aging on the brain relative to the number of traditional chronological aging studies, which compare men and women aged ~65 and older to young adults.

Methods

In March 2019, a survey of the literature was conducted using two search engines, PubMed (PM) and Web of Science (WoS). Our search included articles published between 1995 (corresponding to the rise and subsequent widespread use of functional MRI) and 2017 (the most recent year to be fully indexed).

Reproductive Aging Literature

To quantify the number of neuroimaging papers on reproductive aging, the key word "menopause" was paired with "fMRI", "MRI", "PET", "TMS", "EEG" or "DSI". Across PM and WoS, 1,589 total articles were identified from the key word searches. After filtering for redundancy, these articles were examined further to determine whether they met inclusion criteria. Excluded from consideration were: review articles, abstracts, conference proceedings, animal studies, and articles on the effects of chemically induced hormone suppression if it occurred outside of the midlife period (ages 45-55). If menopause or reproductive aging was mentioned in an article but was not the basis of the study, the article was also excluded. Behavior-only studies were also not considered in the present analysis.

A total of 82 neuroimaging articles of menopause were identified. Articles were then coded for publication year, title, journal, first and last author, sex of the authors, and institution name. Demographic information of the subject cohort was also coded, including sex of the participants (only female or both female and male), mean age and age-range of the sample, the type of menopause studied (spontaneous, surgical, or chemical), the neuroimaging method, and whether the paper reported the use of endocrine assays to confirm menopausal status.

Of the 82 articles identified, 48% were functional magnetic resonance imaging (fMRI) studies, 22% were magnetic resonance imaging (MRI) studies, 16% were positron emission tomography (PET) studies, 11% were electroencephalography (EEG) studies, 2% were spectroscopy studies, and 1% included diffusion spectrum imaging (DSI) as a neuroimaging method in the study. There were no identified reproductive aging articles that reported using transcranial magnetic stimulation (TMS) (See **Supplementary Figure 1**).

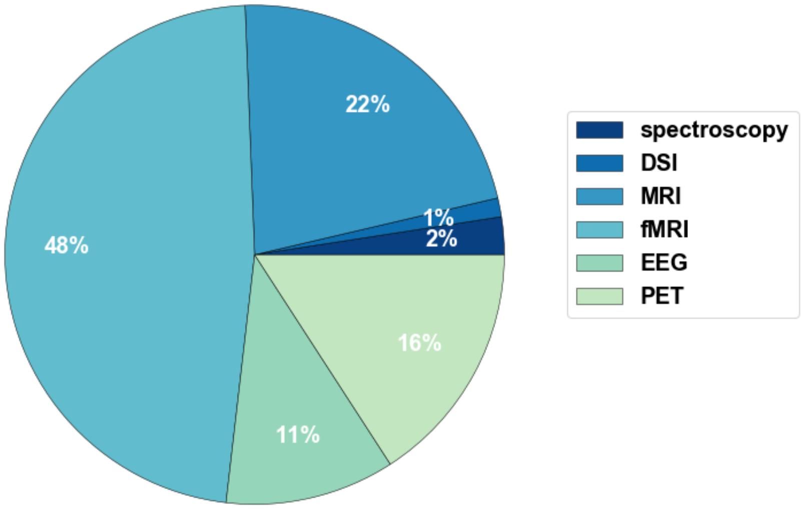

Figure S1. Proportion of neuroimaging methods represented in the reproductive aging literature from 1995 – 2017.

We also quantified how studies verified menopausal stage. Forty-nine percent of the identified articles used serum or saliva-based endocrine assessments, and 51% of the articles used medical histories and participant self-reports, reported that their staging criteria were referenced in previous published studies, or did not report any staging criteria (See **Supplementary Figure 2**).

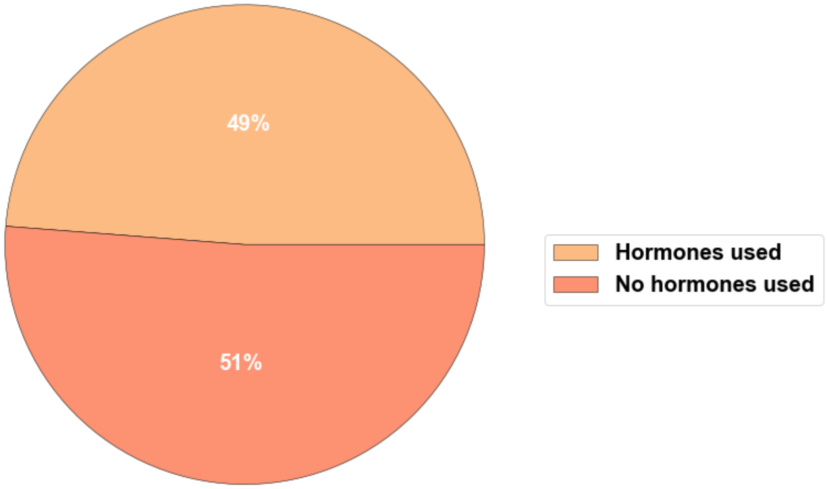

Figure S2. Proportion of reproductive aging articles that used endocrine assessments to verify menopause staging.

Chronological Aging Literature

To provide an estimated number of neuroimaging studies of chronological aging, we created an advanced search criterion in PM in which the key word “aging” was paired with “brain”, “neuroscience”, “cognitive”, or “cognition” and further paired with neuroimaging key words “fMRI”, “MRI”, “PET”, “TMS”, “EEG”, “spectroscopy” or “DSI”. We applied additional filters to PM search results, such that article type was set to “journal article”, text availability was set to “full text”, publication dates were set to “from 1995/01/01 to 2017/12/31”, species was set to “humans”, and ages was set to “aged: 65+ years”. At first pass, the results yielded >125,000 articles. To reduce the results to articles relevant to cognitive neuroscience of aging, case studies, abstracts, reviews, clinical reports, and cancer studies were excluded from this sample. To do this, additional search criteria were applied, using the key words “case study”, “abstract”, “review”, “clinical”, and “cancer” to remove articles that pertain to or mention these as key words, while the key word “human” was added to restrict results to human cognitive aging studies. This search generated 35,071 results, which were downloaded as a .csv file listing article counts per year between 1995 and 2017.

To provide an estimate of the relevant article count within these results, the first 500 articles (of the 35,071) were surveyed to assess how many articles met the inclusionary criteria for neuroimaging studies of chronological aging. Excluded from consideration were: review articles, abstracts, conference proceedings, and animal studies not caught by the previous filter. Eleven percent of the first 500 results fit these criteria. The majority of excluded articles were omitted because they were not neuroimaging studies, were case studies, or review articles. This proportion was then applied to the yearly total search results in order to estimate the number of neuroimaging articles of chronological aging per year of our search window. The number of articles was rounded to the nearest whole number and totaled ~3,929 articles across the 23 year period examined. This corresponds with findings reported by Cabeza and colleagues (2016), who, to illustrate the growing field of cognitive aging within neuroscience, performed a database search for publications related to fMRI of aging and cognition from 1998-2013. From this, they reported that there are thousands of published studies of aging using fMRI and that this number has risen dramatically in the last ten years.

While the tools available for building custom and advanced search criteria within PM are extensive, it is important to note that the estimated number of chronological aging articles represented here may be an underestimation of the total number of neuroimaging articles on cognitive aging. Using Boolean operators to exclude studies with key words such as “review,” for instance, may eliminate relevant citations in the chronological aging search that discuss and reference review articles in the text. Despite this, the estimated number we report here speaks to the fact that, over the last 25 years, the vast majority of chronological aging studies have overlooked women’s reproductive health during the midlife period (Figure 1).

Reference

Cabeza, R., Nyberg, L., and Park, D. (2016) Introduction In R. Cabeza, L. Nyberg, D. Park (eds.) *Cognitive Neuroscience of Aging: Linking Cognitive and Cerebral Aging*. Oxford University Press.
